# Altered Sigmoid Mucosal Innervation and Mast Cell Proximity to Sensory Nerve Fibers Are Associated With Symptom Severity in Patients With Irritable Bowel Syndrome

**DOI:** 10.1111/nmo.70199

**Published:** 2025-11-02

**Authors:** Pu‐Qing Yuan, Michael Nash, Tao Li, Swapna Mahurkar‐Joshi, Jessica Sohn, Ariela Khandadash, Yvette Taché, Lin Chang

**Affiliations:** ^1^ Digestive Diseases Research Center, Vatche and Tamar Manoukian Division of Digestive Diseases, Department of Medicine, David Geffen School of Medicine University of California at Los Angeles Los Angeles California USA; ^2^ VA Greater Los Angeles Healthcare System Los Angeles California USA; ^3^ G. Oppenheimer Center for Neurobiology of Stress and Resilience, Vatche and Tamar Manoukian Division of Digestive Diseases, Department of Medicine, David Geffen School of Medicine University of California at Los Angeles Los Angeles California USA

**Keywords:** abdominal pain, enteric cholinergic fibers, irritable bowel syndrome, mast cells, substance P, vasoactive intestinal peptide

## Abstract

**Background:**

Peripheral neuronal pathways contribute to the pathophysiology of irritable bowel syndrome (IBS).

**Aims:**

To use high‐resolution three‐dimensional (3D) imaging and computerized quantification to compare the density and proximity of distinct nerve fibers (NFs) to mast cells (MCs) in sigmoid mucosal biopsies from patients with constipation‐predominant IBS (IBS‐C), diarrhea‐predominant IBS (IBS‐D), and healthy controls (HCs).

**Methods:**

Sigmoid biopsies from 23 IBS patients (10 IBS‐C [6 females]; 13 IBS‐D [7 females]) and 12 HCs (6 females) were processed, NFs and MCs were immunostained and 3D images were analyzed using Imaris software to quantify NF density and proximity to MCs. Data were correlated with the IBS Severity Scoring System (IBS‐SSS) and abdominal pain ratings.

**Results:**

Compared to HCs, IBS patients had reduced densities of human peripheral choline acetyltransferase (hpChAT, *p* = 0.01) and calbindin (Calb, *p* = 0.08) NFs by 39% and 17%, respectively, with the most prominent reductions of 50% (*p* = 0.004) and 26% (*p* = 0.03) in IBS‐C. Vasoactive intestinal peptide (VIP) NF density was significantly higher in IBS‐D compared to IBS‐C (*p* = 0.01). Other NF densities and MC count did not significantly differ between IBS and HCs. IBS‐SSS and abdominal pain scores were positively correlated with the proximity of MCs to SP (*p* = 0.038) and Calb (*p* = 0.047) NFs and negatively with the density of VIP (*p* = 0.039) and NPY (*p* = 0.005) NFs.

**Conclusions & Inferences:**

In IBS, there are reduced densities of colonic enteric cholinergic and intrinsic primary afferent NFs. Correlations between abdominal pain severity and MC‐NF interactions in IBS patients suggest that altered neuroimmune signaling may contribute to IBS pathophysiology and represent a potential therapeutic target.

## Graphical abstract

3D imaging of nerve fibers and mast cells double‐labeled with antibodies against Substance P (SP, red) and tryptase (green) on CLARITY‐cleared human sigmoid colonic mucosal biopsies from an IBS patient. (A). Their spatial proximity was computationally quantified using Imaris 9.7 software (B), which demonstrated a significant positive correlation with IBS‐SSS (C).

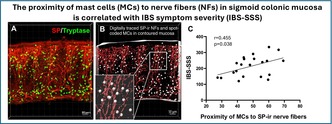

**Summary**

- 3‐dimensional (3D) imaging and computerized quantitation of sigmoid mucosal biopsies showed a decrease in mucosal enteric cholinergic and intrinsic primary afferent nerve fibers in IBS patients compared to healthy controls.
- IBS‐D had a greater density of vasoactive intestinal peptide nerve fibers than IBS‐C.
- Overall, IBS and abdominal pain severity scores were positively correlated with the proximity of mucosal mast cells to substance P and calbindin nerve fibers.
- These findings are indicative of neuroanatomical and neuroimmune alterations in the sigmoid mucosa of IBS patients, involving enteric cholinergic, sensory and secretomotor function that are linked to clinical symptom burden.

## 1 Introduction

Irritable bowel syndrome (IBS) is characterized by recurrent abdominal pain associated with altered bowel habits such as predominant constipation (IBS‐C), diarrhea (IBS‐D), or mixed bowel habits (IBS‐M) [1, 2]. The pathogenesis of IBS is multifactorial and complex, involving the dysregulation between the central and peripheral nervous systems along with brain‐gut interactions [3, 4, 5, 6]. While there are many subtypes of nerve fibers (NFs) that may contribute to IBS pathogenesis [7], two major subtypes are the enteric cholinergic nerves, which regulate intestinal motility among other functions [8], and intrinsic primary afferent nerves, which sense luminal contents and orchestrate intestinal secretion and absorption [9]. In addition, the recruitment and function of immune cells in relation to enteric nerves are thought to contribute to the pathogenesis of IBS through many plausible mechanisms including sensitization of NFs and direct inflammation of colonic tissue [10]. Mast cells (MCs) were reported in close proximity to NFs in the descending colon of IBS patients and their cross‐talk is thought to contribute to visceral pain [11, 12].

NFs, enteric glial cells (EGCs), and immune cells form a three‐dimensional (3D) meshwork in the colonic mucosal lamina propria that cannot be easily portrayed and precisely analyzed with two‐dimensional (2D) images. However, 3D imaging of these long NFs and their spatial configurations with various cell populations, particularly MCs in the whole colonic mucosa, is challenging due to the opacity of the tissue, promoting light scattering and restricting large volume imaging at microscopic resolution. Novel clearing methods revolutionized 3D imaging in the nervous system by increasing visualization at a high resolution [13]. This approach can be combined with immunostaining to targets of interest [14]. Recently, we have developed CLARITY protocols applicable for clearing and 3D imaging of full‐thickness human and pig colon samples [15, 16] and sigmoid mucosal biopsies from healthy controls (HCs) [17, 18]. We have also been able to trace digitally and quantify the density of NFs, MCs, EGCs and the shortest distances of each individual MC to NFs in 3D images using Imaris 9.7–9.9 for Neuroscientists, a software for 3D/4D visualization and segmentation [18]. A detailed visualization of the architecture and topography of this network in its 3D spatial environment will advance our understanding of nerve innervation‐function relationships in the pathophysiology of IBS.

This study aimed to: (1) compare 3D neuronal circuitries, specifically NF densities and MC proximity to NFs, in sigmoid mucosal biopsies from male and female patients with IBS‐D and IBS‐C and HCs and (2) determine the correlation of NF density and MC proximity to NFs with the severity of overall IBS symptoms and abdominal pain.

## 2 Materials and Methods

### 2.1 Study Participants

Male and female HCs and IBS patients aged 18–55 years old were recruited primarily by community advertisement. IBS patients with a bowel habit subtype of IBS‐C or IBS‐D were diagnosed according to the Rome IV criteria [19]. HCs had no personal or family history of IBS or other chronic pain conditions. Additional exclusion criteria for all subjects included infectious or inflammatory disorders, active psychiatric illness over the past 6 months assessed by The Mini‐International Neuropsychiatric Interview (M.I.N.I.) for the Diagnostic and Statistical Manual of Mental Disorders‐IV (DSM‐IV) [20], use of corticosteroids in the past 6 months, narcotics, antidepressants, or a history of current tobacco or alcohol abuse over the same period. Among medications used that may influence GI symptoms or function, two IBS participants used omeprazole 3 months prior to the biopsy date, two IBS participants used loperamide prior to the procedure date (1 day and 6 weeks), one IBS patient used polyethylene glycol and methylcellulose 2 months prior to the procedure, and one participant used psyllium 1 week prior to the procedure. Due to the relatively low number of participants recording use of these medications and lack of known impact of these agents on colonic structure, these participants were retained in the study. None of the participants were taking antispasmodics or consuming a low fermentable oligo‐, di‐, monosaccharide and polyols (FODMAP) diet. Participants were compensated. This study was approved by the UCLA Institutional Review Board (IRB#20‐001727), and all subjects signed a written informed consent form prior to starting the study. Detailed information regarding the characteristics of participants is summarized in Table 1.

**TABLE 1:** Characteristics of study participants.

	All IBS (*N* = 23)	IBS‐C (*N* = 10)	IBS‐D (*N* = 13)	HCs (*N* = 12)
Age [mean (SD)]	32.48 (10.09)	33.50 (10.19)	31.69 (10.36)	34.17 (11.88)
BMI [mean (SD)]	25.02 (4.39)	23.72 (3.35)	25.85 (4.91)	23.54 (3.11)
Sex [count (%F)]	12 (52)	6 (60)	6 (46)	6 (50)
Race [count (%)]				
White	13 (56)	7 (70)	6 (46)	5 (42)
Black	2 (9)	1 (10)	1 (8)	1 (8)
Asian	3 (13)	1 (10)	2 (15)	3 (25)
Multiracial	2 (9)	0 (0)	2 (15)	2 (17)
Declined to state	3 (13)	1 (10)	2 (15)	1 (8)
Ethnicity (count [%Hispanics])	5 (22)	2 (20)	3 (23)	2 (17)
IBS‐SSS (mean (SD), range: 0–500)	216.05 (63.72)	218.1 (60.24)	214.33 (69.10)	—
IBS‐SSS Abdominal Pain Component Subscore (mean (SD), range: 1–100)	32.18 (17.09)	26.7 (19.98)	36.75 (13.44)	—
Abdominal symptom intensity over past 24 h (mean (SD), range: 0–20)	8.32 (4.54)	8.40 (4.14)	8.25 (5.03)	—
Abdominal symptom unpleasantness over past 24 h (mean (SD), range: 0–20)	7.5 (3.14)	7.3 (2.91)	7.3 (2.91)	—

*Note:* Data are presented as mean ± standard deviation (SD) or as a percent as denoted in each row.

Abbreviations: BMI, body mass index; IBS, irritable bowel syndrome; IBS‐C, constipation‐predominant IBS, IBS‐D, diarrhea‐predominant IBS; SD, standard deviation.

### 2.2 Assessment of IBS Symptom Severity

The IBS Severity Scoring System (IBS‐SSS) was used to assess IBS symptom severity [21]. This validated questionnaire measures the frequency and severity of abdominal pain, severity of abdominal distention, dissatisfaction with bowel habits, and interference of IBS with daily life over a 10‐day period. It is scored from 0 to 100 for each of these categories with a total score range of 0–500 and scoring cutoffs indicating the overall symptom severity: > 75–174: mild, > 175–299: moderate, > 300: severe [22]. Current abdominal pain scores including abdominal pain intensity within 24 h of biopsies were measured using a validated numeric rating scale of pain (0–20, 0 indicates no pain and 20 indicates the most intense pain) [23], which has been used in prior IBS studies [24, 25].

### 2.3 Sigmoidoscopy With Biopsies

All participants underwent a flexible sigmoidoscopy procedure with sigmoid mucosal biopsies collected at 30 cm from the anal verge, similar to our previous studies [26, 27]. Tap water enemas were used for cleansing the sigmoid colon on the morning of the procedure. All participants were instructed to withhold taking aspirin and nonsteroidal anti‐inflammatory drugs for 72 h before the procedure. Four sigmoid colonic mucosal biopsies were collected from each participant using jumbo biopsy forceps and processed for the CLARITY tissue clearing procedure. The interval time between the biopsy collection and the beginning of CLARITY was about 60–90 min.

### 2.4 Tissue Clearing

The passive CLARITY protocol of sigmoid mucosal biopsies has been detailed previously [17, 18]. Briefly, the biopsy samples with full thickness of mucosa (sample volume is about 6 × 3 × 1–2 mm length × width × thickness respectively) were rinsed in saline, flattened on cardboard with mucosa facing up and fixed with ice‐cold 4% paraformaldehyde (Sigma‐Aldrich, St Louis, MO) in phosphate‐buffered saline (PBS). The samples were then processed for clearing following steps and reagents as detailed in protocol.io: https://www.protocols.io/view/a‐protocol‐for‐tissue‐clearing‐and‐three‐dimension‐6qpvr8dkblmk/v2. After clearing, samples were immersed overnight in 20% sucrose in PBS for cryoprotection and subsequently embedded in optimal cutting temperature compound (OCT, Thermo Fisher Scientific, Waltham, MA) on dry ice. OCT frozen blocks with cleared samples were vertically sectioned (200‐μm) using a cryostat (Leica CM3050 S, Leica Microsystems Nussloch GmbH, Nussloch, Germany) at −20°C.

### 2.5 Immunohistochemistry

Free‐floating Immunohistochemistry was performed as described previously [17, 18]. Marker antibodies (Table S1) include protein gene product 9.5 (PGP9.5) for pan‐nerve fibers [28], tyrosine hydroxylase (TH) for extrinsic sympathetic nerve fibers [29], neuropeptide (NPY) for sympathetic nerve fibers with both extrinsic and intrinsic origins [30, 31], substance P (SP) mainly for extrinsic primary afferent fibers [32], calbindin (Calb) for intrinsic primary afferent nerve fibers [33], vasoactive intestinal peptide (VIP) for intrinsic secretomotor nerve fibers [33], vesicular acetylcholine transporter (VAChT) for cholinergic nerve fibers with extrinsic and intrinsic origin [34], human peripheral choline acetyltransferase (hpChAT) for intrinsic cholinergic nerve fibers [35, 36], S100β for EGCs [37], and tryptase for colonic mucosal MCs [38]. The primary antibodies were selected based on previous characterization and validation for human colonic samples processed by different methods including the CLARITY tissue clearing technique (https://sparc.science/datasets/290/version/1). Double immunolabeling of PGP9.5, SP or calbindin with tryptase, and VAChT with hpChAT, and single immunolabeling of TH, VIP, NPY, and S100β were conducted by incubating with primary antibodies and fluorochrome‐labeled secondary antibodies [17, 18]. Negative control samples were subjected to the same procedures without the primary antibody. Samples were then immersed in a custom‐made refractive index matching solution (RIMS) and mounted in a sealed watertight well prepared with iSpacers (SunJin Lab, Hsinchu City, Taiwan) which are made from variable thickness adhesive tape [17, 18].

### 2.6 3D Imaging and Quantification

Methods and specifications used for 3D imaging and quantification have been previously published by our group [18] but are partially reproduced here for reference. Z‐stack images with 708 × 708 μm frame (20 × objective) were acquired from CLARITY‐cleared samples with confocal microscopes [18]. Z‐axis intervals were 1 μm with depths of 150–200 μm. 3D images were reconstructed using Imaris 9.7–9.9 for Neuroscientists (Bitplane Inc., Concord, MA). 5–6 3D images of marker‐labeled NFs, EGCs and MCs generated from each participant were quantitated with a computerized approach we published previously [18]. A step‐by‐step computational workflow of this approach has been shared [39]. The density of each subclassified NF was calculated and expressed as the percentage of its volume in the mucosa volume (v/v, %). The density of EGCs was measured as described previously [18] and expressed as the percentage of EGC volume in the mucosa volume (v/v, %).

MCs were spotted with ImarisSpot function and automatically counted with Imaris 9.7–9.9, then density and distances to each NF calculated as reported previously [18]. MC density was calculated and expressed as the number of spots per volume of mucosa (MCs/μm^3^). The shortest distances of the centers of each individual MC spot to the surfaces of PGP9.5‐, SP‐ and Calb‐marked NFs in 3D images were automatically measured and plotted correspondingly with Imaris 9.7–9.9. The analysis was conducted in a blind manner to the experimental performers, and the information on participants (HCs, IBS‐C, IBS‐D, age, and sex) was withheld until after all experiments and quantitation were completed.

### 2.7 Statistical Analysis

Participant characteristics were summarized using means and standard deviations for continuous variables and frequencies and percentages for categorical data. The data of the nerve fiber densities are expressed as means and standard errors of the means (SEM). General linear models (GLMs) were applied to identify differences in the density of NFs, EGCs, and MCs, as well as the proximity of MCs to NFs, between IBS and HCs, between IBS bowel habit subtypes, and between bowel habit subtypes and HCs. P‐values were corrected for multiple comparisons (for the number of markers tested) using false discovery rate (FDR). Additionally, in a sensitivity analysis, IBS vs. HC group differences were assessed using sex as a covariate. The associations between the density of NFs, EGCs, MCs, or the proximity of MCs to the NFs with IBS‐SSS, IBS‐SSS abdominal pain subscore, or current abdominal pain scores including abdominal pain intensity within 24 h of the procedure were performed using Pearson correlation analysis or the framework of GLMs covarying for sex. A *p* value < 0.05 was considered the threshold for reporting.

## 3 Results

### 3.1 Characteristics of Study Participants

The characteristics of the 35 participants including 23 patients with IBS (43% IBS‐C, 57% IBS‐D) and 12 HCs are described in Table 1. There were no statistical differences in sex, age, body mass index (BMI), race or ethnicity between IBS patients and HCs. Overall, IBS symptom severity was moderate with a mean IBS‐SSS total score of 216.1 ± 63.7 and a mean IBS‐SSS abdominal pain subscore of 32.2 ± 17.1. Values were similar between IBS‐C and IBS‐D.

### 3.2 Intrinsic Cholinergic NF Density Is Decreased in IBS Compared to HCs

**FIGURE 1:**
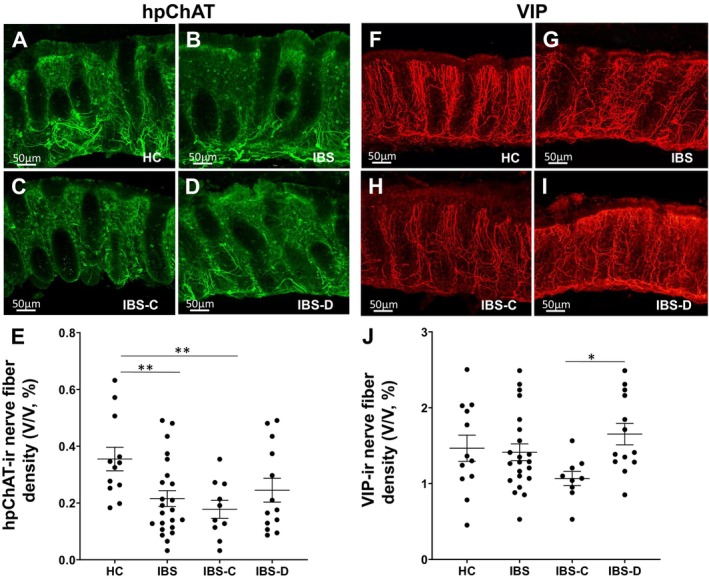
Reduced hpChAT immunoreactive (ir) nerve fiber densities in IBS patients, particularly in IBS‐C, and lower VIP‐ir nerve fiber density in IBS‐C vs. IBS‐D. Images of hpChAT‐ir (A–D) and VIP‐ir (F–I) nerve fibers were created with Imaris slide mode by superimposing about 150–200 Z‐optical slices generated from CLARITY cleared sigmoid mucosal biopsies. (A and F) Healthy controls (HCs). (B and G) IBS patients. (C and H) Constipation‐predominant (IBS‐C). (D and I) Diarrhea‐predominant (IBS‐D). (E and J) The hpChAT‐ and VIP‐ir nerve fiber densities were quantitated in 3D images with a computerized approach we developed. 5–6 of 3D images from each participant were measured and averaged. The data were scatter‐plotted with the individual value of each subject, respectively. The group mean was calculated and expressed as mean ± SEM. *p < 0.05, **p < 0.01 vs. HCs or IBS‐C.

The density of total NFs labeled with the pan‐neuronal marker PGP9.5 in the sigmoid colonic mucosa was not different based on disease group (HCs and IBS), bowel habit (IBS‐C and IBS‐D), or sex, although differences in subtypes of NFs were seen (Tables S2–, S4). However, the intrinsic cholinergic NFs labeled by hpChAT were overall decreased in IBS compared to HCs (Figure 1A,B,E) and were more prominent in IBS‐C (Figure 1C,E). This difference was significant after controlling for sex as a covariate (*p* = 0.008). The density of hpChAT‐labeled NFs was decreased by 39% in the sigmoid mucosa of IBS patients compared to HCs (*p* = 0.007) with a 50% reduction in IBS‐C compared to HCs (*p* = 0.004) and 31% in IBS‐D compared to HCs (*p* = 0.076) (Figure 1E, Tables S2 and S3). In contrast, the density of VChAT‐labeled NFs, which primarily reflect extrinsic cholinergic innervation [34], and sympathetic NFs labeled by TH‐ and NPY in the sigmoid mucosa were similar between HCs and both IBS and IBS bowel habit subtypes (Tables S2 and S3).

### 3.3 Decrease of Calb and VIP NF Densities in IBS Compared to HCs

The intrinsic primary afferent NFs labeled by Calb [33] were reduced in IBS (−17%, *p* = 0.08) and IBS‐C (−26%, *p* = 0.03), but not in IBS‐D, compared to HCs (Tables S2 and S3). The density of NFs marked with VIP, used to label intrinsic secretomotor NFs [33], was reduced 1.5‐fold in IBS‐C compared to IBS‐D (*p* = 0.01) (Figure 1F–J). Other types of NFs labeled by SP and cells labeled by tryptase or S100 ß did not show significant differences in densities between HCs and IBS, IBS‐C and IBS‐D (Tables S2 and S3).

### 3.4 Densities of Mast Cells and Enteric Glial Cells in IBS and HCs

In the sigmoid colonic mucosa, there were no significant differences with regard to the densities of MCs and EGCs labeled with tryptase and S100β, respectively, between HCs and IBS, IBS‐C or IBS‐D (Tables S2 and S3). Double labeling of tryptase with PGP9.5, SP or Calb displayed the spatial configuration of MCs with total and specific NFs and most of the MCs were close to or in contact with PGP9.5, SP or Calb NFs from all directions in the mucosa as shown previously [18].

### 3.5 Digital Tracing and Proximity Measurements of HFs and MCs in IBS and HCs

**FIGURE 2:**
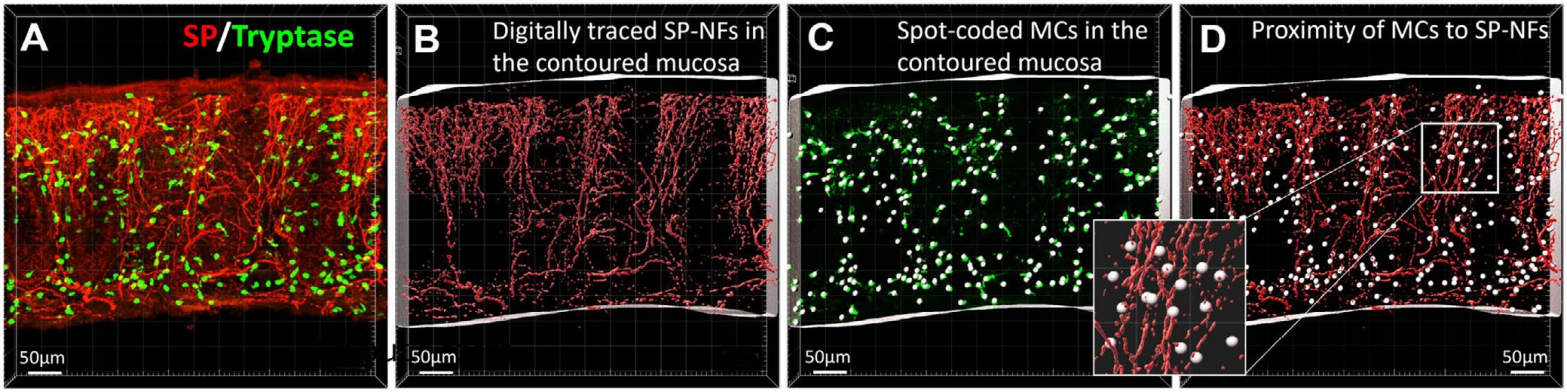
Computational quantitation of nerve fibers (NFs), mast cells (MCs) and the proximity of MCs to the NFs in the human sigmoid colonic mucosal biopsy. (A) A 3D image of a human sigmoid colonic mucosal biopsy generated from Z‐stack confocal images. Sensory nerve fibers (NFs) and mast cells (MCs) were labeled simultaneously by substance P (SP, red) and tryptase (green) antibodies. (B) Digitally traced SP‐NFs in contoured mucosa with ImarisSurface function were used for quantitation of NF density. (C) Spot‐coded MCs in contoured mucosa with ImarisSpot function were used for quantitation of MC density. (D) Digital images were used for automatic measurement of the shortest distances of the centers of each individual spot (MCs) to the surfaces of SP‐NFs in 3D using Imaris 9.7. A spot with the shortest distance equal or less than 5.2 μm to the surface of the fiber was defined as contact to NF on the basis of the sizes of human colonic mucosal MCs with an average diameter of 10.4 μm measured in present study. The proximity of MCs to SP NFs was expressed as a percentage of all MCs in contact to SP NFs in total MCs.

Using ImarisSurface and ImarisSpot functions, the NFs, like those labeled by SP (Figure 2A) were digitally segmented by creating surfaces as red color over them (Figure 2B,D) while the MCs were marked in spots as white color in the centers of each individual MC in 3D images (Figure 2C,D). The entire mucosa was outlined and separated from the submucosa in each 3D image (Figure 2B–D). No significant differences were shown between HCs and IBS, IBS‐C or IBS‐D with regards to the proximity of MCs to total NFs (MC‐PGP9.5), extrinsic primary afferent NFs and intrinsic primary afferent NFs (Tables S2 and S3).

### 3.6 Proximity of MCs to NFs Correlated With Overall IBS Symptoms and Abdominal Pain Severity

**FIGURE 3:**
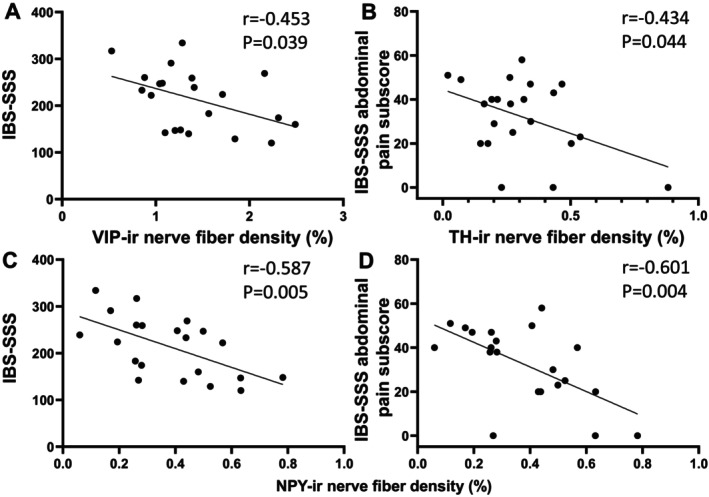
Negative correlation of VIP, TH and NPY immunoreactive (ir) nerve fiber density with IBS‐SSS and IBS‐SSS abdominal pain component subscore in all IBS patients including constipation‐predominant (IBS‐C) and diarrhea‐predominant (IBS‐D). Pearson's correlation coefficients (*r*) were calculated to describe the strength and direction of an association between variables. A *p* value < 0.05 was considered a statistically significant correlation. VIP‐ir: Vasoactive intestinal peptide immunoreactive. TH‐ir: Tyrosine hydroxylase immunoreactive. NPY‐ir: Neuropeptide Y immunoreactive. (A, C) VIP‐ir and NPY‐ir nerve fiber density with IBS‐SSS. (B, D) TH‐ir and NPY‐ir nerve fiber density with IBS‐SSS abdominal pain subscore.

The relationships between mucosal nerve fibers, mast cell proximity, and symptom severity were examined in patients with IBS and the IBS bowel habit subtypes. In all IBS participants, the densities of VIP and NPY NFs were significantly negatively correlated with IBS‐SSS (*r* = −0.453, *p* = 0.039 and *r* = −0.587, *p* = 0.005, respectively) and TH and NPY NFs with the IBS‐SSS abdominal pain component subscore (*r* = −0.434, *p* = 0.044 and *r* = −0.601, *p* = 0.04) (Figure 3A–D; Table S5).

**FIGURE 4:**
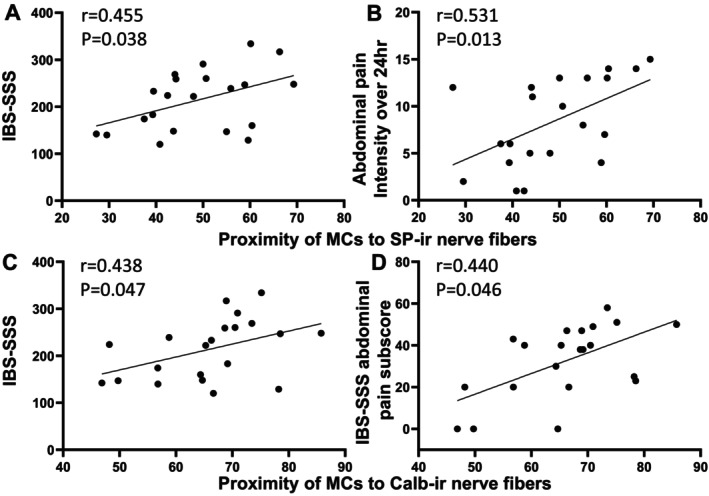
Positive correlation of proximity of mast cells (MCs) to SP‐ and Calb immunoreactive (ir) nerve fibers with IBS‐SSS, IBS‐SSS abdominal pain component subscore or abdominal pain intensity over the 24 h prior to the sigmoidoscopy in IBS patients. Pearson's correlation coefficients (*r*) were calculated to describe the strength and direction of an association between variables. A *p* value < 0.05 was considered a statistically significant correlation. SP‐ir: Substance P immunoreactive. Calb‐ir: Calbindin immunoreactive. (A, B) Proximity of MCs to SP‐ir nerve fibers with IBS‐SSS total score and abdominal pain intensity over 24 h. (C, D) Proximity of MCs to Calb‐ir nerve fibers with IBS‐SSS total score and its abdominal pain subscore.

The proximity of MCs to SP (MC‐SP) and Calb NFs (MC‐Calb) was significantly positively correlated with IBS‐SSS (*r* = 0.455, *p* = 0.038 and *r* = 0.438, *p* = 0.047, respectively) (Figure 4A,C) (Table S5). With respect to abdominal pain, MC‐SP and MC‐Calb positively correlated with abdominal pain intensity over the 24 h before the sigmoidoscopy (*r* = 0.531, *p* = 0.013). In addition, MC‐Calb positively correlated with the IBS abdominal pain subscore (*r* = 0.440, *p* = 0.046) (Figure 4B,D) (Table S5).

**FIGURE 5:**
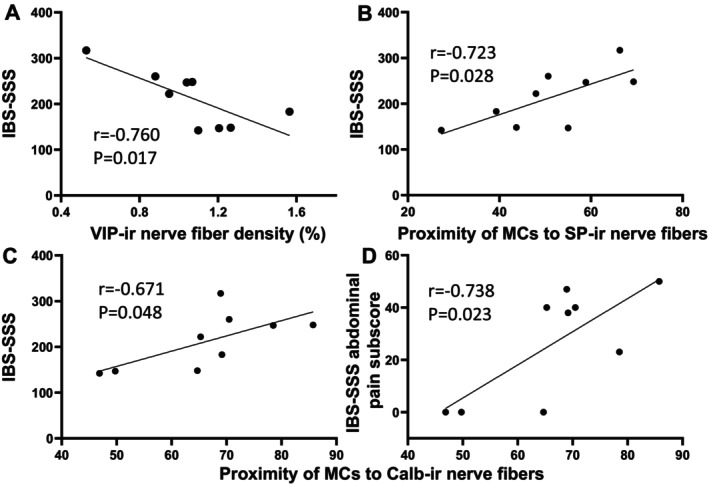
Negative correlation of VIP immunoreactive (ir) nerve fiber density with IBS‐SSS and positive correlation of proximity of MCs to SP‐ and Calb‐ir nerve fibers with IBS‐SSS or IBS‐SSS abdominal pain component subscore in IBS‐C patients. Pearson's correlation coefficients (*r*) were calculated to describe the strength and direction of an association between variables. A *p* value < 0.05 was considered a statistically significant correlation. SP‐ir: Substance P immunoreactive. Calb‐ir: calbindin immunoreactive. (A–C) VIP‐ir nerve fiber density, proximity of MCs to SP‐ir or Calb‐ir nerve fibers with IBS‐SSS. (D) Proximity of MCs to Calb‐ir nerve fibers with IBS‐SSS abdominal pain subscore.

**FIGURE 6:**
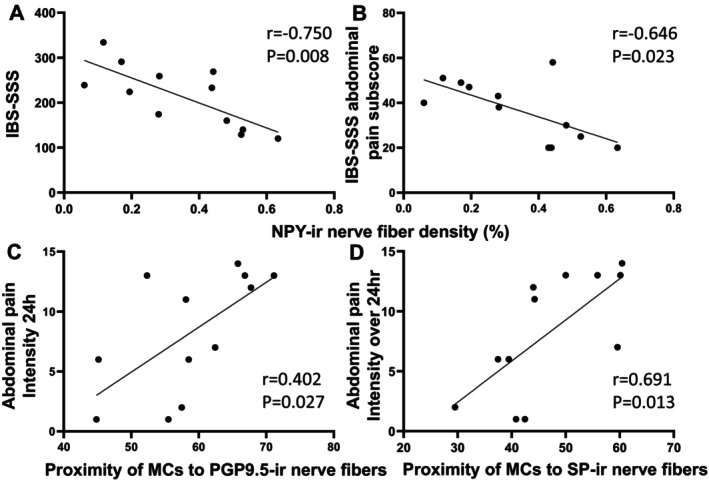
Negative correlation of NPY nerve fiber density with IBS‐SSS and IBS‐SSS abdominal pain component subscore, and positive correlation of proximity of MCs to PGP9.5‐ir and SP‐ir nerve fibers with abdominal pain intensity over the 24 h prior to the sigmoidoscopy in IBS‐D patients. Pearson's correlation coefficients (*r*) were calculated to describe the strength and direction of an association between variables. A *p* value < 0.05 was considered a statistically significant correlation. (A, B) NPY nerve fiber density was negatively correlated with IBS‐SSS total score and its abdominal pain subscore. (C, D) The proximity of MCs to PGP9.5 and SP‐ir nerve fibers displayed positive correlations with abdominal pain intensity over 24 h.

Similar correlations were seen in the IBS bowel habit subtypes. In IBS‐C, the density of VIP‐ labeled NFs was negatively correlated with IBS‐SSS scores. (*r* = −0.760, *p* = 0.017; Figure 5A). MC‐SP and MC‐Calb were positively correlated with IBS‐SSS (*r* = 0.723, *p* = 0.028 and *r* = 0.671, *p* = 0.048, respectively) (Figure 5B,C). MC‐Calb was positively correlated with IBS‐SSS abdominal pain subscore (*r* = 0.738, *p* = 0.023) (Figure 5D). In IBS‐D, the density of NPY‐labeled NFs was negatively correlated with IBS‐SSS scores and abdominal pain subscores (*r* = −0.750, *p* = 0.008 and *r* = 0.646, p = 0.023) (Figure 6A,B). PGP9.5‐MC and MC‐SP were positively correlated with abdominal pain intensity for the 24 h (*r* = 0.402, *p* = 0.027 and *r* = −0.691, *p* = 0.013, respectively; Figure 6C,D). There were no other correlations with the remaining NF subtypes and IBS symptoms or abdominal pain severity (Table S5).

## 4 Discussion

In this study, we used the newly described method of advanced spatial imaging of human colonic biopsies [18] to uncover a reduction in enteric cholinergic nerves and intrinsic primary afferent nerves in IBS, which appeared to be more prominent in IBS‐C. Importantly, the densities of VIP and NPY‐labeled NFs were negatively correlated with IBS symptoms, that is, a higher density of these NFs was associated with lower overall IBS symptom and abdominal pain severity, while greater MC proximity to SP and Calb NFs was associated with increased IBS symptom and abdominal pain severity. Our data support that reductions in colonic intrinsic enteric NF density and increased sensory NF interactions with MCs may underlie IBS symptoms, including abdominal pain, and suggest novel structural differences in the colonic mucosa of patients with IBS compared to HCs.

### 4.1 Implications of Enteric Cholinergic and Intrinsic Primary Afferent NF Density Reductions in IBS Symptoms

To our knowledge, this study is the first to demonstrate reduced densities of enteric cholinergic and intrinsic primary afferent NFs labeled with specific markers hpChAT [34, 35] and Calb [32] in the colonic mucosa of IBS, which appeared to be more prominent in IBS‐C than IBS‐D, using a novel 3D imaging technique. Cholinergic signaling in the colonic mucosa primarily activates epithelial secretion and mucus release and coordinates secretion with motility through a submucosal secretomotor circuit contributing to constipation in IBS‐C [40]. Reduced cholinergic activity has broadly been associated with constipation [7]. Furthermore, higher acetylcholine breakdown in IBS‐D patients is thought to be associated with reduced inhibition of inflammation, which may worsen hyperalgesia in IBS or disrupt gut barrier integrity leading to disruption of colonic secretory function [41]. Peters et al. demonstrated that IBS‐C sigmoid colon biopsies did not have an altered secretory response to acetylcholine in vitro [42]. Thus, reduced mucosal cholinergic (hpChAT) and intrinsic primary afferent (Calb) NFs may be a unique structural feature of IBS‐C that may have a bearing on the manifestation of IBS‐C through reduced colonic secretomotor function and impaired defecation reflex. Calb is a 28 kDa calcium‐binding protein expressed in subsets of enteric neurons, which are actively involved in enteric neurotransmission and may influence gut motility and sensitivity [43]. While direct evidence linking Calb to IBS is limited, our previous study examining gene expression profiles in the sigmoid colonic mucosa of IBS patients showed a significant downregulation of gene expression of CALB2 encoding calb2, a 29 kDa calcium‐binding protein with 58% homology to Calb [44] in IBS patients compared to HCs. Specifically, we found that CALB2 expression was reduced with a fold change of 0.6 (*p* = 0.04) in IBS‐C vs. HCs [27]. Calb, being a key regulator of calcium homeostasis, may be implicated in the altered neuronal excitability seen in IBS patients. An imbalance in calcium regulation through reduced Calb‐labeled NFs could contribute to abnormal secretory motor patterns, defecation reflex and hypersensitivity, leading to symptoms like abdominal pain and bloating [45].

### 4.2 Comparing and Contrasting Other Findings Related to the Density of NFs in IBS

While our work demonstrated reductions in NF densities in IBS, previous reports have shown an overall increase in NFs [46], or increases in subsets of NFs expressing the 5‐HT_7_ serotonin receptor, associated with visceral hypersensitivity [47] in the colonic mucosa of IBS patients. Importantly, these studies did not subdivide phenotypes of NFs as we did, and samples were taken at different biopsy sites (descending colon and ascending and descending colon, respectively) than in our study, which exclusively sampled the sigmoid colonic mucosa. Combined with our findings showing a reduction in intrinsic afferent and mucosal intrinsic cholinergic NFs, it is likely that densities of particular subsets of NFs vary across colon regions, and IBS pathogenesis and symptom severity may be more influenced by changes in the abundance of specific NF subsets rather than overall NF density.

### 4.3 Potential Early Origins of Altered NF Density and Function in IBS

The enteric nervous system (ENS) is developed in utero but continues development in postnatal life [48]. Recent reports indicate a genetic susceptibility to developing IBS [49], with some identified genes involved in the early development of the ENS [6, 50]. The complex developmental processes governing colonic innervation involve the intrinsic ENS establishing key steps before integration with the extrinsic ENS [51]. Our finding of reduced intrinsic, but not extrinsic, NF density in IBS suggests that early, durable alterations in intrinsic innervation may contribute to IBS pathogenesis. This is supported by a study in piglets showing that early life adversity increases cholinergic innervation and secretory function of the ENS [52]. Moreover, we and others have demonstrated that early life adversity, for example, abuse and history of mental illness in the household, are well‐established risk factors for IBS [53, 54, 55]. Additionally, early adverse life events are associated with alterations in central pain processing and emotion regulation networks [56]. Together, these observations suggest the need for future studies to investigate whether reduced intrinsic afferent and enteric cholinergic NF densities represent persistent or early structural features of IBS development. Further, establishing the ontogeny of changes in NF development or function in IBS, specifically intrinsic NF density, should be pursued by examining earlier timepoints, not just in adults as was the case in our study.

### 4.4 Possible Mechanisms for the Association of the Proximity of MCs to Sensory NFs With IBS Symptom Severity

Severity of overall IBS symptoms and abdominal pain were positively correlated with the proximity of mucosal MCs to NFs labeled with SP and Calb, markers for extrinsic [57] and intrinsic sensory neurons [32], respectively. SP NF functions in IBS are broad but include transmitting the sensation of visceral pain and triggering local inflammation [58]. Emerging research supports the association between IBS and altered calcium signaling within the ENS, particularly involving alb. Calcium signaling plays a crucial role in neuronal excitability, and disruptions in this pathway have been implicated in IBS hypersensitivity [45]. In IBS, there is compelling evidence for neuroplastic changes in the colonic mucosa characterized by increased NF density and enhanced interactions between MCs and sensory nerve fibers. Dothel et al. demonstrated that mediators released from the intestinal mucosa of IBS patients significantly increase NF density and sprouting, as well as the expression of nerve growth factor (NGF) and its receptor NTRK1, compared to HCs. Mucosal supernatants from IBS patients induced robust neuritogenesis in primary enteric neuron cultures and NGF‐dependent neuronal sprouting in SH‐SY5Y cells, indicating that the IBS mucosal environment actively promotes neuroplasticity through NGF signaling pathways [46]. These findings are consistent with prior evidence of increased MC number [59] and activation [60] in the colonic mucosa of IBS patients, but it has nevertheless been difficult to distinguish the complex interactions that occur between MCs and NFs, particularly at the level of distinct NF phenotypes. Important findings by Barbara et al. showed that MC proximity to NFs correlates with abdominal pain, although the different subtypes of NFs were not identified in the study [11]. With regard to our data, although SP NF densities were not different in IBS vs. HCs, the association between MC proximity to SP NFs and symptom severity supports functional interactions between neuroimmune remodeling and IBS symptom generation. Further studies should interrogate physical or chemical MC‐nociceptive NF interactions to better identify which phenotypes of NFs are well‐suited to target therapeutically and which interactions may have promise for relieving IBS symptoms.

### 4.5 VIP and New Unique Features and Mechanisms of IBS Bowel Habit Subtype

Importantly, our data show increased VIP‐labeled NFs in IBS‐D compared to IBS‐C, but there is no difference between HCs and IBS patients overall. Previous reports showed that IBS patients have increased VIP in blood, sigmoid tissue and colonic MCs, as well as a higher number of MCs expressing VPAC1 (VIP receptor 1), supporting a neuroimmune mechanism in IBS [61, 62]. Furthermore, transcriptomic studies of rectosigmoid mucosa in IBS‐D demonstrate higher VIP expression [63]. As previously mentioned, IBS‐C sigmoid biopsies did not have an altered secretory response to acetylcholine in vitro [42]. Our findings suggest that increased VIP NF density is a unique feature of IBS‐D that may drive pathophysiology through multiple mechanisms, including enhanced neuroimmune crosstalk, altered epithelial barrier, secretory and peristaltic reflexes, intrinsic sensory afferents and microbiome remodeling [63]. VIP NFs were recently shown to modulate microbiome composition through regulating epithelial fucosylation [64]. A reduction in VIP was proposed to reduce *Bifidobacterium* abundance leading to increased susceptibility of Enterococcal colonization [65]. Multiple studies have demonstrated distinct microbiome compositions in IBS‐D compared to HCs and IBS‐C [66, 67]. Moreover, clinical interventions such as electroacupuncture and moxibustion ameliorate symptoms in IBS by reducing mucosal VIP expression, underscoring VIP's functional role in symptom generation [68]. Taken together with our data, the interaction between enteric cholinergic NFs and MCs may be a major mediator of IBS symptoms rather than secretory function per se. Thus, increased VIP NF density may help distinguish IBS‐D from IBS‐C as a mechanistic biomarker, reflecting a bowel habit subtype characterized by exaggerated neuroimmune signaling and altered microbiome interactions, but this needs additional studies. Further investigations may interrogate the VIP receptor and signaling dynamics of IBS‐D and IBS‐C NFs and better establish the role of VIP signaling in driving symptom burden. Though speculative given our small sample size, a plausible mechanism promoting IBS‐C pathogenesis includes increased MC VIP production and subsequent saturation of VIP receptors on a relatively limited pool of VIP NFs, while altered MC‐NF crosstalk may amplify neuroimmune and microbiome profiles between IBS‐D and IBS‐C, helping to explain the microbiome differences between bowel habit subtypes. These findings help support that IBS‐D and IBS‐C represent mechanistically distinct bowel habit subtypes that will require careful targeted therapeutic approaches.

### 4.6 Study Strengths and Limitations

Strengths of this explorative study include the high clinical value of using human colonic biopsies to offer new insights into IBS pathophysiology. Other strengths included the use of a novel 3D imaging technique to identify previously unassessed structural interactions between NF subtypes and MCs and the ability to correlate these highly granular structural findings with validated symptom severity scores from patients. Another valuable element of our study was the use of biopsies from IBS‐C and IBS‐D bowel habit subtypes to attempt to identify unique features of each disease state.

Limitations include a relatively small sample size in this exploratory study, particularly in the IBS bowel habit subgroups. This limits our ability to characterize differences in NF density or other features distinct or shared between IBS bowel habit subtypes. We were also unable to robustly explore sex‐specific differences given the small sample size. However, our study was able to provide effect size estimates which will guide a well‐powered study (see Tables S2 and S3). Another limitation was solely investigating MCs as the immune cell of interest in relation to NF proximity, as various other immune cells including B‐cells and T‐cells have been shown to contribute to IBS pathophysiology and thus would have been missed with our approach [10]. However, this was beyond the scope of our study and further investigation, perhaps with single nuclei RNA‐seq, will be valuable to address these knowledge gaps. Finally, we only studied biopsies from sigmoid mucosa. While this region of the intestine is highly relevant to IBS [69], we were unable to exclude the possibility that the differences in NF subtypes vary depending on colonic location. Further studies should more thoroughly map NFs and the immune cell landscape across a wider region of colonic tissue.

## 5 Conclusions

Our study provides novel evidence of reduced densities of enteric cholinergic and intrinsic primary afferent NFs in the colonic mucosa of IBS patients, alongside distinct associations between MC‐NF interactions and IBS and abdominal pain severity. These findings suggest that altered neuroimmune architecture, involving specific NF subtypes and their crosstalk with MCs, may contribute to IBS pathophysiology and symptoms, including abdominal pain, and could represent potential therapeutic targets. While our exploratory study offers important novel biological insights, larger, confirmatory studies incorporating additional bowel habit subtypes, colonic regions, and immune cell populations are needed to further elucidate the structural and functional mechanisms underlying IBS pathophysiology.

## Author Contributions

**Pu‐Qing Yuan:** writing‐original draft, review and editing manuscript, methodology, formal analysis, data curation, funding acquisition. **Michael Nash:** editing and giving input on the written manuscript. **Tao Li:** methodology, data curation. **Swapna Mahurkar‐Joshi:** formal analysis. **Jessica Sohn:** data curation. **Ariela Khandadash:** data curation. **Yvette Taché:** writing‐review and editing, funding acquisition. **Lin Chang:** writting‐review and editing, methodology, funding acquisition. All authors listed provide approval for publication.

## Conflicts of Interest

The authors declare no conflicts of interest.

## Supporting information


**Table S1:** Immunofluorescence reagents.

**Table S2:** Comparisons of the densities of nerve fibers (NFs), enteric glial cells (EGCs), mast cells (MCs) and proximity of MCs to NFs in the sigmoid colonic mucosa between healthy controls (HCs) and all irritable bowel syndrome (IBS) patients (mean ± SE).

**Table S3:** Comparisons of the densities of nerve fibers (NFs), enteric glial cells (EGCs), mast cells (MCs) and proximity of MCs to NFs in the sigmoid colonic mucosa among healthy controls (HCs), irritable bowel syndrome (IBS) patients with constipation‐predominant (IBS‐C) and diarrhea‐predominant (IBS‐D) (mean ± SE).

**Table S4:** Comparisons of the densities of nerve fibers (NFs), enteric glial cells (EGCs), mast cells (MCs) and proximity of MCs to NFs between men and women in healthy controls (HCs) and irritable bowel syndrome (IBS) patients (mean ± SE).

**Table S5:** Correlations between the densities of nerve fibers (NF), enteric glial cells (EGC), mast cells (MC), or the proximity of mast cells to nerve fibers with IBS Severity Scoring System (IBS‐SSS) or abdominal pain ratings.

## Data Availability

The data that support the findings of this study are available on request from the corresponding author. The data are not publicly available due to privacy or ethical restrictions.
