# Supplementary material for: Altered Sigmoid Mucosal Innervation and Mast Cell Proximity to Sensory Nerve Fibers Are Associated With Symptom Severity in Patients With Irritable Bowel Syndrome
Source: Neurogastroenterol Motil. 2025 Nov 2;37(12):e70199. doi: 10.1111/nmo.70199 (PMC12623276; doi:10.1111/nmo.70199)
Supplement: Supplementary file 1 — Table S1: Immunofluorescence reagents. [file NMO-37-e70199-s005.docx]

|  | **Name** | **Host** | **Source/ Product no.** | **Dilution** |
| --- | --- | --- | --- | --- |
| **Primary antibodies** | Protein gene product 9.5 | Rabbit | Abcam/ ab108986 | 1:1000 |
|  | Tyrosine hydroxylase | Rabbit | Millipore/ AB152 | 1:1000 |
|  | Substance P | Rabbit | ImmunoStar/ 20064 | 1:1000 |
|  | Vasoactive intestinal peptide | Rabbit | CURE UCLA/ 7913 | 1:1000 |
|  | Vesicular acetylcholine transporter | Rabbit | Synaptic System/ 139 103 | 1:1000 |
|  | Human peripheral form of choline acetyltransferase | mouse | Dr. Bellier/ H3 | 1:2000 |
|  | Calbindin | Rabbit | Swant/ CB-38 | 1:1000 |
|  | Neuropeptide | Rabbit | CURE UCLA/ 8713 | 1:500 |
|  | S100β | Rabbit | Abcam/ ab52642 | 1:1000 |
|  | Mast Cell Tryptase, AA1 | Mouse | Agilent Dako/ M7052 | 1:500 |
| **Secondary antibodies** | Alexa 488-conjugated anti-mouse IgG | donkey | ThermoFisher Scientific/A-32766 | 1:400 |
|  | Alexa 555-conjugated anti-rabbit IgG | donkey | ThermoFisher Scientific/A-31572 | 1:400 |
| **Normal serum** | Normal donkey serum | donkey | Jackson ImmunoResearch | 1:10 |

Supplementary Table 1. Immunofluorescence reagents
