# Supplementary material for: Altered Sigmoid Mucosal Innervation and Mast Cell Proximity to Sensory Nerve Fibers Are Associated With Symptom Severity in Patients With Irritable Bowel Syndrome
Source: Neurogastroenterol Motil. 2025 Nov 2;37(12):e70199. doi: 10.1111/nmo.70199 (PMC12623276; doi:10.1111/nmo.70199)
Supplement: Supplementary file 2 — Table S2: Comparisons of the densities of nerve fibers (NFs), enteric glial cells (EGCs), mast cells (MCs) and proximity of MCs to NFs in the sigmoid colonic mucosa between healthy controls (HCs) and all irritable bowel syndrome (IBS) patients (mean ± SE). [file NMO-37-e70199-s001.docx]

|  | HCs n=12 | IBS n=23 | t-statistic | P value | FDR |
| --- | --- | --- | --- | --- | --- |
| ^a^PGP9.5 | 2.432±0.156 | 2.539±0.156 | -0.44 | 0.66 | 0.846 |
| ^a^SP | 0.927±0.121 | 1.130±0.114 | -1.13 | 0.27 | 0.846 |
| ^a^Calb | 1.798±0.135 | 1.488±0.0998 | 2.22 | 0.08 | 0.505 |
| ^a^VIP | 1.466±0.172 | 1.413±0.110 | 0.27 | 0.79 | 0.846 |
| ^a^VAChT | 0.189±0.0385 | 0.211±0.0384 | -0.36 | 0.72 | 0.846 |
| ^a^hpChAT | 0.355±0.0415 | 0.216±0.0278 | 2.86 | **0.01** | 0.143 |
| ^a^TH | 0.340±0.0315 | 0.283±0.0409 | 0.42 | 0.68 | 0.846 |
| ^a^NPY | 0.398±0.0328 | 0.388±0.0398 | 0.17 | 0.87 | 0.846 |
| ^b^S100β | 2.148±0.134 | 2.331±0.101 | -1.08 | 0.29 | 0.846 |
| ^c^Tryptase | 1.56E-05±1.03E-06 | 1.52E-05±7.70E-07 | 0.31 | 0.76 | 0.846 |
| ^d^MC-PGP9.5 | 59.427±3.412 | 62.440±1.896 | -0.84 | 0.41 | 0.846 |
| ^d^MC-SP | 46.898±5.514 | 48.439±2.391 | -0.3 | 0.77 | 0.846 |
| ^d^MC-Calb | 68.612±3.988 | 65.899±2.121 | 0.66 | 0.51 | 0.846 |

**Supplementary Table 2.** Comparisons of the densities of nerve fibers (NFs), enteric glial cells (EGCs), mast cells (MCs) and proximity of MCs to NFs in the sigmoid colonic mucosa between healthy controls (HCs) and all irritable bowel syndrome (IBS) patients (mean±SEM)

Abbreviations: PGP9.5: protein gene product 9.5, SP: substance P, Calb: calbindin, VIP: vasoactive intestinal peptide, VAChT: vesicular acetylcholine transporter, hpChAT: human peripheral choline acetyltransferase, TH: tyrosine hydroxylase, NPY: neuropeptide, MC-PGP9.5, -SP, -Calb: the proximity of mast cells to PGP9.5, SP and Calb nerve fibers. ^a^: NF density (v/v, %), ^b^: EGC density (v/v, %), ^c^: MC density (No. of MCs/µm^3^), ^d^: proximity of MCs to NFs (%). FDR: false discovery rate. The bolded value indicates significant difference.
