# Supplementary material for: Altered Sigmoid Mucosal Innervation and Mast Cell Proximity to Sensory Nerve Fibers Are Associated With Symptom Severity in Patients With Irritable Bowel Syndrome
Source: Neurogastroenterol Motil. 2025 Nov 2;37(12):e70199. doi: 10.1111/nmo.70199 (PMC12623276; doi:10.1111/nmo.70199)
Supplement: Supplementary file 4 — Table S4: Comparisons of the densities of nerve fibers (NFs), enteric glial cells (EGCs), mast cells (MCs) and proximity of MCs to NFs between men and women in healthy controls (HCs) and irritable bowel syndrome (IBS) patients (mean ± SE). [file NMO-37-e70199-s002.docx]

|  | HCs | | IBS | | ANOVA P value | FDR |
| --- | --- | --- | --- | --- | --- | --- |
|  | Men n=6 | Women n=6 | Men n=11 | Women n=12 |  |  |
| ^a^PGP9.5 | 2.453 ±0.223 | 2.411±0.238 | 2.541±0.310 | 2.538±0.115 | 0.979 | 0.979 |
| ^a^SP | 1.000±0.199 | 0.854±0.150 | 1.272±0.137 | 1.012±0.174 | 0.408 | 0.979 |
| ^a^Calb | 2.047±0.114 | 1.549±0.205 | 1.485±0.197 | 1.491±0.077 | 0.095 | 0.6175 |
| ^a^VIP | 1.569±0.308 | 1.363±0.179 | 1.383±0.169 | 1.438±0.151 | 0.912 | 0.979 |
| ^a^VAChT | 0.196±0.067 | 0.183±0.045 | 0.229±0.060 | 0.196 ±0.052 | 0.952 | 0.979 |
| ^a^hpChAT | 0.395±0.070 | 0.315±0.045 | 0.184±0.040^e^ | 0.244±0.039 | **0.029** | 0.377 |
| ^a^TH | 0.317±0.045 | 0.363±0.047 | 0.282 ±0.0501 | 0.346±0.111 | 0.741 | 0.979 |
| ^a^NPY | 0.362±0.045 | 0.434±0.047 | 0.353±0.060 | 0.417 ±0.054 | 0.709 | 0.979 |
| ^b^S100β | 2.100±0.233 | 2.195±0.154 | 2.237 ±0.194 | 2.417±0.079 | 0.575 | 0.979 |
| ^c^Tryptase | 1.58E-05±  1.77E-06 | 1.54E-05±  1.24E-06 | 1.58E-05 ±  1.13E-06 | 1.47E-5 ±  1.07E-06 | 0.902 | 0.979 |
| ^d^MC-PGP9.5 | 61.123±3.847 | 57.730±5.939 | 64.149 ±3.194 | 61.016±2.285 | 0.678 | 0.979 |
| ^d^MC-SP | 50.242±8.537 | 43.555±7.513 | 50.395±3.188 | 46.810±3.542 | 0.793 | 0.979 |
| ^d^MC-Calb | 71.830±6.499 | 65.395 ±4.859 | 66.456 ±2.871 | 65.434 ±3.176 | 0.711 | 0.979 |

Supplementary Table 4. Comparisons of the densities of nerve fibers (NFs), enteric glial cells (EGCs), mast cells (MCs) and proximity of MCs to NFs between men and women in healthy controls (HCs) and irritable bowel syndrome (IBS) patients (mean±SEM)

Abbreviations: PGP9.5: protein gene product 9.5, SP: substance P, Calb: calbindin, VIP: vasoactive intestinal peptide, VAChT: vesicular acetylcholine transporter, hpChAT: human peripheral choline acetyltransferase, TH: tyrosine hydroxylase, NPY: neuropeptide, MC-PGP9.5, -SP, -Calb: the proximity of mast cells to PGP9.5, SP and Calb nerve fibers. ^a^: NF density (v/v, %), ^b^: EGC density (v/v, %), ^c^: MC density (No. of MCs/µm^3^), ^d^: proximity of MCs to NFs (%). ^e^: p=0.024 IBS vs HCs in men. The bolded value indicates significant difference.
