# Supplementary material for: Altered Sigmoid Mucosal Innervation and Mast Cell Proximity to Sensory Nerve Fibers Are Associated With Symptom Severity in Patients With Irritable Bowel Syndrome
Source: Neurogastroenterol Motil. 2025 Nov 2;37(12):e70199. doi: 10.1111/nmo.70199 (PMC12623276; doi:10.1111/nmo.70199)
Supplement: Supplementary file 5 — Table S5: Correlations between the densities of nerve fibers (NF), enteric glial cells (EGC), mast cells (MC), or the proximity of mast cells to nerve fibers with IBS Severity Scoring System (IBS‐SSS) or abdominal pain ratings. [file NMO-37-e70199-s003.docx]

| **IBS (IBS-C+IBS-D)** |  | IBS-SSS | | | IBS-SSS abdominal pain subscore | | | Abdominal pain intensity over 24 hr | | |
| --- | --- | --- | --- | --- | --- | --- | --- | --- | --- | --- |
|  |  | R value | P value | FDR | R value | P value | FDR | R value | P value | FDR |
|  | ^a^PGP9.5 | -0.294 | 0.185 | 0.426 | -0.349 | 0.111 | 0.307 | -0.072 | 0.752 | 0.339 |
|  | ^a^SP | 0.179 | 0.436 | 0.567 | -0.184 | -0.425 | 0.613 | -0.122 | 0.598 | 0.386 |
|  | ^a^Calb | 0.075 | 0.740 | 0.740 | 0.132 | 0.559 | 0.726 | -0.006 | 0.979 | 0.878 |
|  | ^a^VIP | 0.453 | **0.039** | 0.154 | -0.095 | 0.682 | 0.806 | -0.173 | 0.454 | 0.386 |
|  | ^a^VAChT | 0.279 | 0.221 | 0.426 | -0.312 | 0.169 | 0.365 | -0.083 | 0.722 | 0.386 |
|  | ^a^hpChAT | 0.183 | 0.414 | 0.567 | -0.035 | 0.877 | 0.950 | 0.239 | 0.283 | 0.716 |
|  | ^a^TH | 0.222 | 0.321 | 0.522 | -0.434 | **0.044** | 0.200 | -0.169 | 0.451 | 0.339 |
|  | ^a^NPY | 0.609 | **0.005** | 0.067 | -0.611 | **0.004** | 0.052 | -0.443 | 0.063 | **0.001** |
|  | ^b^S100β | 0.267 | 0.229 | 0.426 | -0.207 | 0.354 | 0.613 | -0.178 | 0.427 | 0.339 |
|  | ^c^Tryptase | 0.098 | 0.672 | 0.728 | -0.190 | 0.407 | 0.613 | -0.117 | 0.614 | 0.386 |
|  | ^d^MC-PGP9.5 | 0.130 | 0.574 | 0.679 | 0.014 | 0.954 | 0.954 | 0.324 | 0.152 | 0.716 |
|  | ^d^MC-SP | 0.455 | **0.038** | 0.154 | 0.352 | 0.118 | 0.307 | 0.531 | **0.013** | 0.339 |
|  | ^d^MC-Calb | 0.438 | **0.047** | 0.154 | 0.440 | **0.046** | 0.200 | 0.292 | 0.200 | 0.603 |
| **IBS-C** | ^a^PGP9.5 | 0.508 | 0.134 | 0.350 | -0.528 | 0.117 | 0.400 | -0.158 | 0.663 | 0.780 |
|  | ^a^SP | 0.087 | 0.824 | 0.930 | -0.295 | 0.441 | 0.640 | -0.211 | 0.586 | 0.780 |
|  | ^a^Calb | 0.056 | 0.878 | 0.930 | -0.151 | 0.967 | 0.970 | -0.179 | 0.620 | 0.780 |
|  | ^a^VIP | 0.760 | **0.017** | 0.180 | -0.395 | 0.292 | 0.600 | -0.564 | 0.114 | 0.780 |
|  | ^a^VAChT | 0.093 | 0.812 | 0.930 | -0.328 | 0.390 | 0.630 | -0.386 | 0.304 | 0.780 |
|  | ^a^hpChAT | 0.220 | 0.541 | 0.880 | 0.136 | 0.709 | 0.840 | 0.208 | 0.565 | 0.780 |
|  | ^a^TH | 0.224 | 0.533 | 0.880 | -0.490 | 0.151 | 0.400 | -0.211 | 0.558 | 0.780 |
|  | ^a^NPY | 0.444 | 0.231 | 0.500 | -0.518 | 0.153 | 0.400 | -0.479 | 0.192 | 0.780 |
|  | ^b^S100β | 0.555 | 0.096 | 0.310 | -0.349 | 0.323 | 0.600 | -0.166 | 0.646 | 0.780 |
|  | ^c^Tryptase | 0.197 | 0.612 | 0.890 | -0.255 | 0.508 | 0.660 | -0.475 | 0.196 | 0.780 |
|  | ^d^MC-PGP9.5 | 0.037 | 0.926 | 0.930 | -0.028 | 0.942 | 0.970 | -0.133 | 0.733 | 0.790 |
|  | ^d^MC-SP | 0.723 | **0.028** | 0.180 | 0.557 | 0.120 | 0.400 | 0.369 | 0.328 | 0.780 |
|  | ^d^MC-Calb | 0.670 | **0.048** | 0.210 | 0.738 | **0.023** | 0.300 | 0.067 | 0.864 | 0.860 |
| IBS-D | ^a^PGP9.5 | 0.045 | 0.889 | 0.972 | 0.159 | 0.622 | 0.885 | 0.031 | 0.924 | 0.924 |
|  | ^a^SP | 0.333 | 0.290 | 0.556 | -0.234 | 0.463 | 0.885 | -0.069 | 0.833 | 0.906 |
|  | ^a^Calb | 0.281 | 0.376 | 0.556 | 0.195 | 0.544 | 0.885 | 0.212 | 0.509 | 0.877 |
|  | ^a^VIP | 0.522 | 0.082 | 0.429 | -0.052 | 0.874 | 0.885 | -0.067 | 0.837 | 0.906 |
|  | ^a^VAChT | 0.499 | 0.099 | 0.429 | -0.419 | 0.176 | 0.811 | 0.094 | 0.770 | 0.906 |
|  | ^a^hpChAT | 0.380 | 0.223 | 0.556 | -0.170 | 0.597 | 0.885 | 0.268 | 0.399 | 0.877 |
|  | ^a^TH | 0.274 | 0.389 | 0.556 | -0.195 | 0.544 | 0.885 | -0.166 | 0.607 | 0.877 |
|  | ^a^NPY | 0.750 | **0.008** | 0.101 | -0.646 | **0.023** | 0.300 | -0.459 | 0.133 | 0.589 |
|  | ^b^S100β | 0.011 | 0.972 | 0.972 | 0.047 | 0.885 | 0.885 | -0.202 | 0.530 | 0.877 |
|  | ^c^Tryptase | 0.011 | 0.973 | 0.972 | 0.080 | 0.806 | 0.885 | 0.178 | 0.579 | 0.877 |
|  | ^d^MC-PGP9.5 | 0.268 | 0.399 | 0.556 | 0.409 | 0.187 | 0.811 | 0.402 | **0.027** | 0.175 |
|  | ^d^MC-SP | 0.262 | 0.412 | 0.556 | 0.243 | 0.446 | 0.885 | 0.691 | **0.013** | 0.168 |
|  | ^d^MC-Calb | 0.253 | 0.428 | 0.556 | 0.073 | 0.822 | 0.885 | 0.502 | 0.096 | 0.416 |

**Supplementary Table 5.** Correlations between the densities of nerve fibers (NF), enteric glial cells (EGC), mast cells (MC), or the proximity of mast cells to nerve fibers with IBS Severity Scoring

System (IBS-SSS) or abdominal pain ratings

Abbreviations: IBS: irritable bowel syndrome, IBS-C: constipation-predominant IBS, IBS-D: diarrhea-predominant IBS. PGP9.5: protein gene product 9.5, SP: substance P, Calb: calbindin, VIP: vasoactive intestinal peptide, VAChT: vesicular acetylcholine transporter, hpChAT: human peripheral choline acetyltransferase, TH: tyrosine hydroxylase, NPY: neuropeptide, MC-PGP9.5, -SP, -Calb: the proximity of mast cells to PGP9.5, SP and Calb nerve fibers. ^a^: NF density (v/v, %), ^b^: EGC density (v/v, %), ^c^: MC density (No. of MCs/µm^3^), ^d^: proximity of MCs to NFs (%). FDR: false discovery rate. Bolded values indicate significant correlations.
